# Influencing factors of work stress of medical workers in clinical laboratory during COVID-19 pandemic: Working hours, compensatory leave, job satisfaction

**DOI:** 10.3389/fpubh.2023.1078540

**Published:** 2023-02-03

**Authors:** Gang He, Yongquan Chen, Dai Wang, Houzhao Wang

**Affiliations:** ^1^School of Public Health, Xiamen University, Amoy, Fujian, China; ^2^Department of Clinical Laboratory, Xiang‘an Hospital of Xiamen University, Amoy, Fujian, China

**Keywords:** clinical laboratory, COVID-19, epidemic, work stress, medical workers

## Abstract

**Background:**

The COVID-19 pandemic continues to pose unprecedented threats and challenges to global public health. Hospital Clinical Laboratory and public health institutions have been playing an important role in case detection, epidemic research and decision-making, and epidemic prevention and control.

**Objective:**

To explore the current situation and influencing factors of work stress of medical workers in hospital clinical laboratory in fighting against COVID-19.

**Methods:**

A cluster random sampling method was used to select seven hospitals from 14 tertiary hospitals in Xiamen, and medical workers in the selected hospitals were investigated by self-administered questionnaire. A total of 150 medical workers inclinical laboratory participated in this survey, 138 valid questionnaires were collected, with a response rate of 92%.

**Results:**

The work stress scores of the medical workers in the clinical laboratory of hospital in the COVID-19 epidemic were collected (55.22 ± 11.48); The top three dimensions of work stress score were work stress (work load), external environment and doctor-patient relationship. The results of multiple stepwise regression analysis showed that the working hours per day, whether overtime and night shift can get compensatory leave and Job satisfaction with the work of the clinical laboratory were the main factors affecting the work stress level of medical workers in the clinical laboratory of hospital during COVID-19 epidemic.

**Conclusion:**

The COVID-19 has caused great harm to the physical and mental health of the public. Medical staff are in the front line of prevention and control of the epidemic, so medical workers in hospital clinical laboratory exposed to a high level of stress at work. Laboratory leaders and hospital managers should take active and effective measures to reduce the working hours of the medical staff in clinical laboratory, optimize the arrangement of night shift and overtime working, strengthen the training of group and individual pressure management, reduce the work stress of the medical staff, improve the overall happiness of the medical staff in clinical laboratory, and stabilize the clinical laboratory team, improve the physical and mental health of medical workers in clinical laboratory.

## Introduction

According to the information provided by the World Health Organization, globally, as of June 16, 2022, there have been 535,248,141 confirmed cases of COVID-19, including 6,313,229 deaths, and more than 600,000 new confirmed cases were added every day (1). On January 20, 2020, the National Health Commission of the People's Republic of China incorporated the new coronavirus infection into the Class B infectious diseases stipulated in the “Prevention and Control Law of the People's Republic of China,” and adopted the prevention and control measures of class A infectious diseases (2). There is no doubt that COVID-19 pandemic is a multifaceted macroeconomic shock, which may have a negative long-term impact on the economy in recent years (3). The atmosphere of job insecurity and economic turbulence caused by the COVID-19 are also spreading, which also seriously affects the mental health of citizens (4). Because of the role of work in supporting one's physical and mental health and self-esteem, job insecurity can seriously affect one's self-efficacy, confidence, and social support system (5). The phenomenon of elevated work stress and job insecurity is even more obvious among medical workers, as hospitals have been in the front line of prevention and control since the outbreak of COVID-19. Under the background of China's normalized epidemic prevention and control, the hospital's laboratory has to undertake the work of nucleic acid sample testing from a large number of people in the hospital and the local area while carrying out its daily tasks. Due to the lack of sufficient human resources. Due to the lack of sufficient human resources, It is unavoidable to take more workloads for the hospital's laboratory of the COVID-19. In addition, the infectious disease report, the entry of test results and other operations of the COVID-19 require the hospital to allocate special personnel to be responsible, which makes human resources particularly scarce (6).Moreover, because of the huge risks in the biological safety management of the laboratory, it also puts medical workers in the laboratory department under even greater work pressure (7). The clinical laboratory is also a wind vane for epidemic prevention and control, and the medical staff in the laboratory are an important and special force for the normalization of epidemic prevention and control. If the medical workers of the laboratory work under excessive pressure without corresponding measures to alleviate and improve it, it will not only be detrimental to the work and development of the hospital, but also bring adverse effects to the epidemic prevention and control.

Work stress refers to the tension or threat that the staff feels in the process of engaging in a certain occupation (8). In addition, during the outbreak of COVID-19, health care workers (HCWs) experience significantly higher work stress than the general population, and the increase in work stress was associated with job burnout (9, 10). Kuo et al. (11) conducted a study on the perceived work stress and its influencing factors among hospital staff during the COVID-19 pandemic in Taiwan, China. The research results showed that the reported stress level of hospital staff was medium. The discomfort caused by protective equipment is the main stressor for participants, and the burden of caring for patients is the second job stressor. According to the survey results, the persistence of the COVID-19 epidemic has led to increasing work pressure faced by doctors. In the context of the normalization of epidemic prevention and control, the doctors' job stressors are mainly from the external environment, workload and career development (12).

On the one hand, the indirect impact of COVID-19 related work stress on anxiety and depression through flexibility is far greater than other indirect effects, and the COVID-19 pandemic has a significant psychological effect on Italian general practitioners and health care workers (HCWs) (13, 14). Zhang et al. (15) conducted a cross-sectional survey of frontline medical workers fighting against COVID-19 in Wuhan, Harbin and Shenzhen, and showed that burnout was common among frontline medical personnel, and that work stress and inadequate protection affected frontline medical personnel's burnout symptoms. Na and Tao et al. (16, 17) found that mental health problems and burnout were prevalent among medical workers in the clinical laboratory, and psychological state and burnout were correlated, and that promoting a reduction in burnout could improve the psychological state of medical workers. On the other hand, in the face of the accelerated pace of life and work, as well as the intensification of social competition, the majority of professional groups are under pressure from different aspects. Although the increase of work stress can improve work efficiency to a certain extent, the increase of work stress will lead to more boredom and physical fatigue to work, and then develop to absenteeism and resignation, the increase of work stress seriously affected physical and mental health, which has become an important public health issue (18). A study found that all HCWs will encounter stress and are frequently exposed to unfavorable conditions in their work and life. The study assessed the relationship between burnout, insomnia, and anxiety severity. The study have found that a bilateral association between insomnia and burnout, so further meaningful intervention measures should be promoted to improve shift work in HCWs (19).

At present, most of the research on staff related to the COVID-19 have focused on front-line nurses, emergency department doctors, primary medical workers and so on. Few studies have paid attention to the work stress situation of medical workers in hospital clinical laboratory under the background of normalized epidemic prevention and control. Our study had two primary objectives. First of all, to provide a reference for hospital managers or health policy makers in order to take practical and feasible measures to ensure the physical and mental health of clinical laboratory medical workers, stabilize the working status of the hospital's clinical laboratory; Secondly, in order to stabilize the laboratory team of the hospital, successfully fight against the epidemic, and steadily promote the epidemic prevention and control work, corresponding countermeasures and suggestions are provided for the medical staff in the laboratory.

## Theory: Job demand resource (JDR) model

The selection of key variables in this study is based on the Job Demand Resource (JDR) model, which was originally developed to better understand the effects of job stress, initially focusing on the effects of job engagement (20) and job burnout (21). This model proposes that specific physical, psychological, social or organizational factors are related to work stress (21). The JDR model divides job characteristics into job demands and job resources. Job demands are the “negative factors” that consume an individual's energy at work and are defined as the physical, psychological, social, or organizational demands of the job that require cost or effort on the part of the individual (employee) to complete the work, including work overload, time pressure, job insecurity, etc. Job resources are “positive factors” at work, defined as factors at work that facilitate the achievement of job goals and promote personal learning and development, including social support, job satisfaction, self-efficacy, and general wellbeing, etc.

Job demands and resources directly affect burnout or work engagement, respectively, while the imbalance between job demands and job resources may be a factor contributing to increased work stress. Job resources have a buffering effect to mitigate the adverse effects of job demands on the development of work stress (22). Therefore, we included “working hours per day” and “the availability of compensatory time off for overtime and night shifts” as representative factors of job demands in the questionnaire, and “the job satisfaction” as a representative factor of job resources (23) into the basic information options of the questionnaire. Based on the JDR model and the actual situation, we made a simple descriptive analysis of the work stress of medical workers in clinical laboratory.

## Methods

### Participants and sampling

#### A cross-sectional survey

Using the cluster sampling method, from July 15 to August 1, 2022, seven hospitals were randomly selected from 14 hospitals in Xiamen, and medical workers were selected from clinical laboratories of selected hospitals to carry out the survey. The inclusion criteria were as follows: Inclusion criteria of survey objects: Formal staff in clinical laboratory; Participated in the fight against the COVID-19 epidemic; Volunteer to participate in this research. As of August 1, 2022, the medical workers from hospital clinical laboratory in Xiamen (~150) were invited to participate in this study, with a response rate of 92%, which resulted in a sample of 138 medical workers.

## Measurement

### Basic situation survey form

Based on the literature review and expert consultation (24–30), a self- administered general information and demographic questionnaire was developed, including age, gender, marital status, level of academic qualification, professional qualification, Service years, monthly income, working hours per day, whether the overtime working or night shifts can get compensated leave, and job satisfaction with the work of the clinical laboratory.

### Stressor scale for medical workers in hospital clinical laboratory

Since there is no relatively mature questionnaire on job stressors for medical workers in the clinical laboratory in China, and considering that it is not appropriate to use a questionnaire with too many questions, we finally used Professor Cooper's job stressors scale (31) and the job stressors scale for doctors produced by Chen (32) in China as a model to prepare a work stress questionnaire for medical staff in the hospital clinical laboratory, which was divided into two parts: basic information, work stress status survey.

The scale has good reliability and validity, with a Cronbach's alpha value of 0.86. There are 19 entries divided into seven dimensions: organizational management, vocational interest, workload, career development, doctor-patient relationship, interpersonal relationship, and external environment. Each item was assessed on the Likert 5-point rating scale, ranging from “No stress” to “Tremendous stress,” with a total score of 1–5 for each item, with higher scores indicating greater stress. In our study, scores of 19–38 were classified as mild stress, 39–57 as moderate stress, and 58–95 as severe stress.

### Survey method and data collection

An online survey link or quick response code (by way of the questionnaire website platform) was sent to the relevant heads of the selected hospital's clinical laboratory and they were invited to send it to the medical workers within the department. Participants could complete the questionnaire *via* a computer or smartphone that could open the website link or scan the QR code. The researchers initially explained in detail the purpose and significance of the study, how to ensure participant anonymity and confidentiality, and other relevant information. Participants agreed if they connected to the website link and completed the questionnaire.

### Statistical methods

Statistical data were represented by frequencies and percentages, and measurements were expressed as x ± s. Two independent sample *t*-tests were used for the comparison between the two groups, The comparison between multiple groups adopts one-way ANOVA, and then the multi-linear regression analysis is performed for multi-factor analysis. All statistical analyses were performed with SPSS for Windows 26.0, and a two-sided *p* < 0.05 was considered statistically significant.

## Results

### Basic information

The distribution of demographic data of 138 medical workers in the clinical laboratory of hospital during the COVID-19 pandemic is shown in Table 1.

**Table 1:** Demographic characteristics of study participants (*n* = 138).

**Variable**		**Numbers (*n*)**	**Percentage (%)**
Gender	Male	48	34.78
	Female	90	65.22
Age	<30	41	29.71
	31–40	55	39.86
	41–50	28	20.29
	>50	14	10.14
Marital status	Unmarried	44	31.88
	Married	92	66.67
	Divorced or widowed	2	1.45
Level of academic qualification	Junior college or below	21	15.22
	Undergraduate	84	60.87
	Master	30	21.74
	Doctor	3	2.17
Professional qualification	No qualification yet	10	7.25
	Primary	42	30.43
	Intermediate	52	37.68
	Senior	34	24.64
Service years	<3 years	26	18.84
	3–5 years	23	16.67
	5–10 years	25	18.12
	>10 years	64	46.38
Monthly income (yuan)	<6,000	13	9.42
	6,000–10,000	47	34.06
	10,001–15,000	51	36.96
	>15,000	27	19.57
Working hours per day	6–8 h	31	22.46
	8–10 h	64	46.38
	10–12 h	43	31.16
Whether the overtime working or night shifts can get compensated leave	Yes	33	23.91
	No	105	76.09
Job satisfaction with the work of the clinical laboratory	Very satisfied	5	3.62
	Relatively satisfied	31	22.46
	Basically satisfied	59	42.75
	Relatively dissatisfied	39	28.26
	Very unsatisfied	4	2.90

### Work stress of medical workers in the clinical laboratory

Under the background of COVID-19, the average work stress score of 138 medical staff in the clinical laboratory of major hospitals in Xiamen is 55.22 ± 11.48, which is a medium stress level. The specific scores of each dimension are shown in Table 2.

**Table 2:** Work stress scores of medical workers in combating the COVID-19 epidemic (χ ± s, *n* = 138).

**Dimension**	**Score**		**Rank**
	**Dimension**	**Entry**	
Organizational management	7.99 ± 2.64	2.66 ± 0.88	5
vocational interest	4.65 ± 1.79	2.33 ± 0.90	6
Work stress and workload	11.36 ± 2.88	3.79 ± 0.96	1
Career development	8.36 ± 2.44	2.79 ± 0.81	4
Interpersonal relationship	6.37 ± 2.09	2.12 ± 0.70	7
Doctor patient relationship	9.96 ± 2.56	3.32 ± 0.85	2
External environment	6.53 ± 1.78	3.26 ± 0.89	3
Total score	55.22 ± 11.48	2.91 ± 0.60

### Single-factor analysis of work stress of medical workers in clinical laboratory

The results showed that Whether the overtime working or night shifts can get compensated leave and Job satisfaction with the work of the clinical laboratory affect the work stress scores of medical staff in clinical laboratory (*p* < 0.05), as shown in Table 3.

**Table 3:** Single-factor analysis of the job stress of medical staff working in the clinical laboratory (χ ± s, *n* = 138).

**Variable**		**Total points**	**Statistics**	***p*-value**
Gender	Male	53.69 ± 10.56	*t* = −1.145	0.283
	Female	56.03 ± 11.91		
Age	<30	56.66 ± 11.83	*F* = 1.175	0.256
	30–39	56.25 ± 11.02		
	40–50	52.61 ± 12.12		
	>50	55.07 ± 10.36		
Marital Status	Unmarried	58.52 ± 10.33	*F* = 1.226	0.205
	Married	53.93 ± 11.69		
	Divorced or widowed	45.50 ± 0.71		
Level of academic qualification	Junior college or below	58.14 ± 11.36	*F* = 0.670	0.929
	Undergraduate	54.27 ± 11.80		
	Master	56.69 ± 10.67		
	Doctor	49.00 ± 8.91		
Professional qualification	No qualification yet	54.00 ± 12.85	*F* = 1.239	0.193
	Primary	56.71 ± 11.77		
	Intermediate	57.69 ± 10.73		
	Senior	49.94 ± 10.49		
Service years	<3 years	59.58 ± 8.22	*F* = 1.218	0.212
	3–5 years	56.43 ± 13.23		
	6–10 years	54.12 ± 9.19		
	>10 years	53.44 ± 12.42		
Monthly income	<6,000	55.15 ± 10.91	*F* = 0.852	0.719
	6,000–10,000	54.68 ± 12.23		
	10001–15000	56.31 ± 11.47		
	>15000	54.11 ± 10.83		
Working hours per day	6–8 h	48.32 ± 13.99	*F* = 1.263	0.173
	8–10 h	54.52 ± 9.50		
	10–12 h	55.22 ± 11.48		
Whether the overtime working or night shifts can get compensated leave	Yes	45.73 ± 12.33	*t* = −2.538	0.003
	No	58.20 ± 9.44		
Job satisfaction with the work of the clinical laboratory	Very satisfied	41.20 ± 20.01	*F* = 2.308	0.000
	Relatively satisfied	49.16 ± 11.16		
	Basically satisfied	54.15 ± 10.23		
	Relatively dissatisfied	62.33 ± 7.18		
	Very unsatisfied	66.00 ± 4.24		

### Multi-factor analysis of work stress among medical workers in the laboratory department

The work stress scores of medical staff in hospital clinical laboratory were the dependent variable, with the single-factor analysis of their stress load, and the titles of basic information were used as independent variables for stepwise multiple linear regression analysis. The results showed that working hours per day, whether the overtime working or night shifts can get compensated leave and Job satisfaction with the work of the clinical laboratory were the main factors influencing the job stress levels of nurses assisting in the fight against COVID-19, which explains 49% of the total variance, as shown in Table 4.

**Table 4:** Multiple-factor analysis of the job stress of Medical staff working in the clinical laboratory.

**Dependent variable**	**Regression coefficient**	**SE**	**Standardized regression coefficient**	***t*-value**	***p*-value**
Constant	22.034	5.895	3.681	0.000
Working hours per day	3.205	1.089	0.192	2.778	0.006
Whether the overtime working or night shifts can get compensated leave	9.147	1.913	0.341	4.782	0.000
Job satisfaction with the work of the clinical laboratory	4.704	0.895	0.360	5.253	0.000

*R* = 0.700, *R*^2^ = 0.490, *F* = 12.203, *P* < 0.01.

## Discussion

In past few years many medical advancements have been made in theoretical epidemiology (33) and practical medicine (34–37) that contribute to innovation and development of medical science (38). After the outbreak of COVID-19 epidemic, the majority of clinical laboratory workers worked day and night in the front line of clinical work to complete the detection of SARS-CoV-2 specific nucleic acids and antibodies, and the medical staff was already close to overload due to the increasingly heavy workload, tedious and repetitive work tasks, isolation periods of varying lengths, and various pressures brought about by the work-family conflict (39). According to the on-site survey, the medical staff of the clinical laboratory doctors work intensely, there is no concept of holidays. On average, each medical personnel have a night shift as well as many overtime shifts; More than 90% of the medical staff of the clinical laboratory feel psychological pressure and high work stress. The main stress comes from the working duration, the amount of work tasks, because the working hours per day and per week had an impact on work stress (40, 41). The results of our study found that the work stress of 138 medical workers participating in the survey had a single mean score of 2.91 ± 0.60 for work stress, which was generally moderate. The three dimensions with the highest scores in each dimension are work stress and workload, doctor-patient relationship and external environment, which is consistent with the survey results of Mo et al. (40). The research shows that work stress is negatively related to work capacity, work stress and presenteeism have a significantly adverse impact on task performance of medical staff, and health status has a enormous positive impact on task performance (42, 43). COVID-19 pandemic has not only led to a series of changes in working methods, but also had a great impact on individual psychological wellbeing (44). When dealing with the challenges of panic and fear caused by the COVID-19, psychosocial support and effective organizational support systems are crucial, an open working environment, stress management training and strong social ties can help employees effectively cope with the crisis and reduce work stress (45). COVID-19 has caused serious economic uncertainty and potential economic pressure, factors related to work stress will have a negative impact on the working environment, employee performance and work efficiency will also be reduced. However, managers can take measures to actively respond, improve the safety awareness of employees during the epidemic, ensure business continuity to reduce job insecurity, improve the job satisfaction of employee, and reduce the pressure and anxiety of employee (46, 47). Therefore, we suggest that hospital managers should pay more attention to the work stress of medical workers in clinical laboratory of the hospital, and take positive and effective measures to improve the working environment of the clinical laboratory, optimize the work content of the department staff, reasonably adjust the working hours, and ensure the physical and mental health of medical workers.

### Factors influencing the work stress of medical staff in the laboratory of hospitals in fighting against COVID-19

The results showed that the working hours per day, whether the overtime working or night shifts can get compensated leave and Job satisfaction with the work of the clinical laboratory were the main factors affecting work stress of medical staff in clinical laboratory of the hospital in fighting COVID-19.

#### Daily working hours

This study found that daily working hours were positively related to work pressure. the longer the working hours, the more stressful the work of medical staff was. Since the global outbreak of COVID-19 at the end of December 2019, Xiamen has experienced several epidemic crises. As the front-line staff for epidemic prevention and control, the medical staff in clinical laboratory are required to wear protective clothing, N95 masks, goggles, rubber gloves and other protective props due to the particularity of the work content, which increases the inconvenience of daily work. Working in a closed environment for a long time will lead to an increase in the working stress level (40).The data showed that working 40–50 h per week were positively associated with the work efficiency (48) without considering the work input. The work environment was significantly associated with self-rated health status and working hours. The risk of poor health status was greater for staff who had longer working hours and with higher education (49). Under the background of the COVID-19 epidemic, the working environment of the hospital's clinical laboratory is relatively closed, the atmosphere is slightly suppressed, the medical workers have long working time, and the work stress and physical and mental pressure are tremendous.

Whether the overtime working or night shifts can get compensated leave: our results show that if the overtime working or night shifts can get compensated leave, and the work stress of medical workers in the hospital clinical laboratory will be reduced accordingly. Research shows that there is an association between a high proportion of night shift work and long-term chronic diseases that can cause health problems. Higher rates of sick leave associated with long night shifts may result in additional costs or lost productivity for hospitals (50). Working long hours does not directly affect mental health; However, it will indirectly affect psychosomatic stress reaction and depressive symptoms through factors such as shortened sleep time and irregular meal time (51). Working hours are significantly related to psychological stress response. Workers are prone to show higher psychological stress response when working overtime on weekdays and holidays. Regardless of the type of work, reducing overtime is very important for reducing the psychological stress reaction (52). The workers who worked overtime for a long time showed significantly higher “irritability,” “fatigue,” “anxiety,” “depression” and “somatic reaction” in both genders. The overtime was linearly related to various stress reactions. There was a linear relationship between the length of overtime and various psychosomatic stress responses (53). At the same time, medical workers' alertness and work performance remain the worst during night shifts because of the difficulty of adapting to circadian rhythms (54). Clinical laboratory medical workers are also required to respond to public health emergencies or unexpected medical events during the night shift, which will lead to greater work pressure on medical staff in the clinical laboratory.

#### Job satisfaction with the work of the clinical laboratory

Our findings showed that medical workers who are less satisfied with clinical laboratory work also have a higher level of work stress. Data research showed that occupational burnout, anxiety, and depression are common among medical workers. When facing work stress, it is more likely to lead to job burnout and work satisfaction of medical workers (55). In addition, an analysis of data has shown that job dissatisfaction is positively related to depression, anxiety and work stress (56, 57). Staffing, work environment, and working hours are significantly associated with nurse burnout and job dissatisfaction (58). Also, social support is positively associated with job satisfaction (59). Social support and work stress were negatively correlated, and the reinforcement of social support can reduce the work stress of medical workers (60). The job satisfaction of medical staff not only has a direct negative impact on turnover intention, but also has an indirect impact on turnover intention through work engagement (61). Stabilizing the work team, building a favorable working environment, and making rational work arrangements are crucial for the development of departments and even hospitals.

### Implications for hospital administrators as well as clinical laboratory leaders

The leaders of the clinical laboratory leaders and hospital administrators should pay attention to the impact of work stress on medical workers in the laboratory department, take active and effective measures to eliminate hospital laboratory staff stressors, unite the medical staff team, and steadily solid the work of epidemic prevention and control. Research shows that adequate sleep, the company of family and pets, and good communication between teams are effective coping styles for job stressors (62). Psychological training and group consultation can effectively reduce job stress, improve self-efficacy and staff confidence (63). Work life balance is of great significance to job satisfaction. Providing a good working environment and facilities, implementing a fair salary and remuneration system and establishing a good communication system in the department can well-improve the work enthusiasm of employees and reduce the work stress of staff (64, 65). The organization can reduce the workload of staff and reduce role conflict and role ambiguity by rationalizing shifts and working hours and optimizing shift arrangements, thus reducing the stress of staff. In addition, the organization should also introduce employee assistance programs and stress management technologies, provide consulting services for employees, help employees learn stress management skills, and conduct stress management, so as to improve their work enthusiasm and productivity (47). Therefore, the hospital can create a harmonious and appropriate working environment through the salary and bonus incentive system, reasonable arrangement of working hours, optimization of shift arrangement, employee assistance plan, group and individual stress management training, optimization of the layout of department work areas, and improvement of department communication system, which will help alleviate the working stress of medical staff in front-line laboratory under the background of normalized epidemic prevention and control, Improve the work quality and efficiency of the hospital laboratory. Hospitals should create a harmonious and appropriate working environment through salary and bonus incentive system, reasonable arrangement of working hours and shifts, group and individual training, and optimization of department work area layout, which will help relieve the working stress of medical workers in front-line clinical laboratory under the background of normalized epidemic prevention and control.

## Strengths and limitations

We are the first known study conducted in a municipal administrative region of China on the work stress of a specific group of medical workers in hospital clinical laboratories, which makes our research valuable. In addition, the survey we conducted adopts cluster random sampling method. Compared with ordinary convenient sampling, our research samples are more random and more representative of the research population. And it should be noted that the study has the following limitations. First, due to human, financial and time constraints, this survey was conducted by selecting only a special group of medical workers in the clinical laboratory of hospitals that experienced COVID-19 pandemic, which also resulted in an insufficient number of samples that could be collected by the questionnaire. Therefore, the results of our study data are not particularly satisfactory. Secondly, because this study is a cross-sectional design, we only evaluated the working pressure at a specific time without longitudinal observation, and some potential influencing factors of working stress may not be taken into account. Therefore, it is necessary to expand the research scope and include more potential factors affecting work stress in future research.

## Conclusion

COVID-19 pandemic has caused an unprecedented huge impact on all countries in the world. The clinical laboratory plays a particularly important role in the disease management of the COVID-19. The clinical laboratory is crucial to early detection of cases and prevention of disease transmission (66). During the epidemic of COVID-19, the laboratory of domestic hospitals made great contributions to the prevention and control of the epidemic in china, and the work stress of the laboratory of Chinese hospitals deserves attention. In our study, the medical staff in the hospital laboratory are generally under high working stress. The main factors affecting the working stress of the medical staff in the hospital clinical laboratory are working hours per day, whether overtime and night shift can get compensatory leave and Job satisfaction with the work of the clinical laboratory. At the same time, we put forward some countermeasures and suggestions to alleviate the work stress, which also provides reference for other countries to adjust the work stress, maintain physical and mental health of the corresponding medical staff, and improve public health.

## Data availability statement

The raw data supporting the conclusions of this article will be made available by the authors, without undue reservation.

## Ethics statement

The studies involving human participants were reviewed and approved by Medical Ethics Committee of Xiang'an Hospital, Xiamen University. The patients/participants provided their written informed consent to participate in this study.

## Author contributions

GH and YC conceived the study, designed and collected questionnaires, and performed the statistic analysis. GH created and performed the literature search strategy and wrote the original draft. YC and DW guided GH to revise the manuscript. YC, DW, and HW reviewed the manuscript and supervised the process. All authors have read and approved the final manuscript.
